# Utility of adjuvant whole abdominal radiation therapy in ovarian clear cell cancer (OCCC): a pragmatic cohort study of women with classic immuno-phenotypic signature

**DOI:** 10.1186/s13014-021-01750-4

**Published:** 2021-02-06

**Authors:** Mark J. Stevens, Simon West, Gregory Gard, Christopher Renaud, David Nevell, Stephanie Roderick, Andrew Le

**Affiliations:** 1 Department of Radiation Oncology, Northern Sydney Cancer Centre, Royal North Shore Hospital, Level 1 ASB Building, St Leonards, NSW 2065 Australia; 2 Northern Clinical School, University of Sydney, St Leonards, NSW Australia; 3 Department of Obstetrics and Gynecology, Royal North Shore Hospital, St Leonards, NSW Australia; 4 Department of Pathology, Royal North Shore Hospital, St Leonards, NSW Australia

**Keywords:** Clear cell ovarian cancer, Intensity-modulated radiation therapy, Whole abdominal radiation therapy, Hepatocyte nuclear factor (HNF)

## Abstract

**Background:**

To evaluate the initial experience and clinical utility of first-line adjuvant intensity-modulated whole abdominal radiation therapy (WART) in women with ovarian clear cell cancer (OCCC) referred to an academic center.

**Methods:**

Progression-free and overall survival was analyzed in a pragmatic observational cohort study of histologically pure OCCC patients over-expressing HNF-1ß treated between 2013 and end-December 2018. An in-house intensity-modulated WART program was developed from a published pre-clinical model. Radiation dose-volume data was curated to American Association of Physics in Medicine (AAPM) Task Group 263 recommendations. A dedicated database prospectively recorded presenting characteristics and outcomes in a standardized fashion.

**Results:**

Five women with FIGO (2018) stage IA to IIIA2 OCCC were treated with first-line WART. Median age was 58 years (range 47–68 years). At diagnosis CA-125 was elevated in 4 cases (median 56 kU/L: range 18.4–370 kU/L) before primary de-bulking surgery. Severe premorbid endometriosis was documented in 3 patients. At a median follow-up of 77 months (range 16–83 mo.), all patients remain alive and progression-free on clinical, biochemical (CA-125), and ^18^Fluoro-deoxyglucose (FDG) PET/CT re-evaluation. Late radiation toxicity was significant (G3) in 1 case who required a limited bowel resection and chronic nutritional support at 9 months post-WART; 2 further patients had asymptomatic (G2) osteoporotic fragility fractures of axial skeleton at 12 months post-radiation treated with anti-resorptive agents (denosumab).

**Conclusions:**

The clinical utility of intensity-modulated WART in OCCC over-expressing HNF-1β was suggested in this small observational cohort study. The hypothesis that HNF-1β is a portent of platinum-resistance and an important predictive biomarker in OCCC needs further confirmation. Curating multi-institutional cohort studies utilizing WART by means of “Big Data” may improve OCCC care standards in the future.

## Background

According to the International Agency for Research on Cancer (IARC) GLOBOSCAN 2018 report, ovarian cancer affects mainly younger women (78% cases < 70 years) and has a relatively poor survival with 5-year death rates of over 50 percent [1, 2]. It is estimated that the global burden of ovarian cancer will increase 47% from 2018 to 2040 (295,414 to 434,184 women). Without new methods of prevention, early diagnosis, and treatment, the IARC projects that the overall mortality of ovarian cancer world-wide will rise nearly 59% with the future deaths of an extra 108,240 women (total estimated 293,039) throughout the 5 continents over the next 20 years. Globally, ovarian cancer will remain the second leading cause of gynecologic cancer mortality [1–3].

Primary cancers of surface-epithelial origin constitute the majority (90%) of ovarian malignancies [4]. Although these all originate from the primitive Mullerian epithelium, considerable heterogeneity in biologic behavior, genetic stability, therapeutic responsiveness, and mortality risk [5–12] exist within the 4 major epithelial phenotypes: serous, mucinous, endometrioid, and clear cell cancer. Paradoxically, despite the known disparities between these subtypes, current therapeutic algorithms for epithelial ovarian cancer remain largely harmonious with primary or secondary de-bulking surgery followed or preceded (respectively), by platinum/taxane-based chemotherapy.

Ovarian clear cell cancer (OCCC) is the most recently characterized and least common of the epithelial histologies in North American and European women (overall prevalence 4–9.5% [3, 6, 33]). It is arguably the most lethal subtype even when detected early [3–6, 8–14]. Occurring in younger cohorts [2, 9] it is particularly aggressive when locally advanced (extra-pelvic peritoneal or lymph node dissemination), or recurrent [4, 15, 16]. Strongly associated with precursor endometriosis [17], Asian ethnicity (prevalence in Japanese cancer registries can exceed 40% [13]), and with unique genotypic and phenotypic signatures, the prognosis of OCCC is generally regarded as dismal due to inherent resistance to platinum-based chemotherapy [10, 16]. Globally there is an exigency for a reliable adjuvant treatment for women with OCCC.

Overexpression of the master transcription factor, hepatocyte-nuclear-factor 1beta (HNF-1β) is a diagnostic marker of OCCC. Current evidence suggests that HNF-1β is a major contributory or interactive [6, 18] driver of platinum resistance and thus also predictive of OCCC outcome.

Recently [3, 6], the disappointing efficacy of platinum-based regimens in OCCC has re-activated interest in the historical strategy of first-line adjuvant whole abdominal radiation therapy (WART) after optimal de-bulking surgery. But concerns remain over the ultimate efficacy and fidelity of this complex radiation technique, and the potential for severe normal tissue toxicity using earlier protocols and radiation technologies [3, 19–26, 39, 42].

We describe for the first time the clinical utility of contemporary intensity-modulated WART in a small prospective series of OCCC patients whose phenotypic signature included HNF-1β over-expression. It was hypothesized that modern WART would be a safe and non-futile therapeutic strategy in women predicted to be platinum-resistant.

## Methods

This study was pre-approved by the Northern Sydney Local Health District (NSLHD) and included patients who had consented to the use of their oncologic and general medical data stored by the Northern Sydney Cancer Center for clinical follow-up and research purposes.

We prospectively assessed and recorded on a dedicated RT database the cancer outcomes and emergent radiation toxicity of all women receiving first-line adjuvant WART following comprehensive primary staging surgery for OCCC over-expressing HNF-1β. Five women were identified with OCCC between January 2013 and end December 2018 and all had prior resection to minimal residual disease by a single gynecologic oncologist (GG). Table 1 summarizes the cohort demographics, tumor characteristics, and International Federation of Gynecology and Obstetrics (FIGO) 2018 staging. All histopathology was re-interpreted by a specialist gynecologic pathology service (CR and DN). WART was recommended for this OCCC cohort after Tumor Board discussion.

**Table 1:** Patient demographics and staging characteristics

Patient	Age (years)	[Ca-125] (kU/L)	Stage (2018)	Follow-up (months)	Peritoneal cytology	Endometriosis (years)	Ascites	Uni-lateral or bilateral tumors	Maximum weight (gms)
1	68	370	IC1 (T1c1)	83	Negative	No	No	Bilateral	910/194
2	65	167	IIA (T2a)	81	Positive	No	No	Unilateral	347
3	48	18	IIIA2 (T3a2)	77	Positive	Yes (25 years)	No	Unilateral*	75
4	47	48	IA (T1a)	57	Negative	Yes (25 years)	No	Unilateral	369
5	58	56	IC3 (T1c3)	16	Positive	Yes (30 years)	No	Unilateral	111

^*^Prior adnexectomy

A diagnosis of classic OCCC was recorded if > 90% of the examined operative specimen demonstrated typical histo-morphological features, such as cells with abundant clear to granular eosinophilic cytoplasm, arranged in tubulo-cystic, complex papillary, or solid architecture. OCCC arising from, or contiguous with, co-existent endometriosis or atypical endometrioid progenitors was also documented. For the purpose of this analysis, initial OCCC morphologic tissue descriptors were re-validated and curated with a quantitative OCCC immuno-histochemical (IHC) signature panel [31]: namely, positive for HNF-1β and P53-wildtype; negative for both Wilm’s tumor suppressor (WT-1) antibody, and estrogen receptor-alpha subunit (ER-α). Phenotypic harmony within the WART treatment group was noted (Table 2). For completeness, the retrospective IHC panel also documented: AMACR (alpha methyl Acyl-CoA racemase), Napsin-A, and loss of expression of the tumor suppressor gene AT-rich interaction domain 1A (ARID1A). Immuno-staining was performed with an automated stainer [Benchmark XT (Ventana) for the ER stain, BOND-III (Leica) for all others]. Monoclonal primary antibodies were used for WT-1 (WT 49), ER (SP1), P53 (D07), and AMACR (EPMU1). Rabbit polyclonal primary antibodies were used for HNF-1β, Napsin-A, and ARID1a. All staining was performed at the Department of Anatomical Pathology, Royal North Shore Hospital, Sydney.

**Table 2:** OCCC immuno-phenotypic characteristics according to Remmele and Stegner [27]

Patient	WT-1	ER $\alpha$	p53	HNF1-ß*	AMACR**	NAPSIN-A	ARID1A^¶^
1	Moderate	Negative	Normal	Moderate	Negative	Negative	Positive
2	Negative	Negative	Normal	Strong	Moderate	Weak	Positive
3	Negative	Negative	Normal	Strong	Weak	Negative	Negative
4	Negative	Negative	Normal	Strong	Strong	Moderate	Positive
5	Negative	Negative	Normal	Strong	Moderate	Negative	Negative

*Hepatocyte nuclear factor-1ß

** Alpha methyl Acyl-CoA racemase

AT-rich interaction domain 1A

The IHC stains were evaluated using the immuno-reactive Remmele score (IRS) [27]. ARID1a staining was divided into negative (IRS score 0–2) and positive cases (IRS score 3–12). P53 was divided into normal (IRS score 3–8) and abnormal (IRS 0–2 or 9–12), with both strong, diffuse positivity and complete negativity considered as evidence of a P53 mutation.

### Toxicity

Acute and late (> 90 day) WART-related toxicity data was collected by physician and patient-reported assessments according to the Common Terminology Criteria for Adverse Events (CTCAE v4.03) [28]. Anatomically, 5 CTCAE system organ classes (SOC) were predicted to be affected by WART, namely, blood and lymphatic system, gastrointestinal tract, hepato-biliary system, musculo-skeletal system, and renal and urinary tract. Structured questionnaires and specific SOC-related blood tests (bone marrow and liver function) were administered.

During WART, patients had weekly on-treatment clinical reviews which included history and physical examination and differential full blood count (FBC), and liver and kidney function tests. Post-WART oncologic reviews (history and physical examination, serum [CA-125], and hepato-renal biochemistry) were scheduled at week 6, week 12, and then quarterly for 2 years. Metabolic [FDG (18Fluoro-deoxyglucose) PET] re-staging was repeated on weeks 12, 52, and 104 (i.e. 2-year PET). From beginning of year 3, patients were seen 6-monthly, then annually after year-5. FDG-PET imaging was repeated if clinically indicated at any time.

### WART

Adjuvant WART commenced within 6 weeks of primary de-bulking surgery. An in-house automated dual iso-centre volumetrically modulated arc-based therapy (VMAT) technique was developed from the pre-clinical dosimetric modeling study of Mahantshetty et al. [29].

For clarity in this analysis, we first curated the abdominal and pelvic WART planning target volumes (PTV) as low dose (PTV_Low) and high dose (PTV_High), respectively. Organ-at-risk (OAR) structure names (liver, lungs, heart, kidneys, bladder, and rectum) were also labeled according to the AAPM (American Association of Physicists in Medicine) Task Group 263 formalism [30]. The aim of WART was to synchronously deliver a homogeneous dose of 25 Gy (PTV_Low) and 45 Gy (PTV_High), in 25 daily fractions (over 5 consecutive weeks) utilizing a simultaneous integrated boost to the pelvic peritoneum and lymph nodes (PTV_High) while protecting the OARs as avoidance tissues from significant radiation exposures. This was achieved using inverse optimization (Progressive Resolution Optimizer v11.0.31, Photon Optimizer v15.6.03 Varian Medical Systems, USA) and calculated using the Anisotropic Analytical Algorithm v11.0.31, v15.6.03 Varian Medical Systems, USA).

### Process

**Fig. 1:**
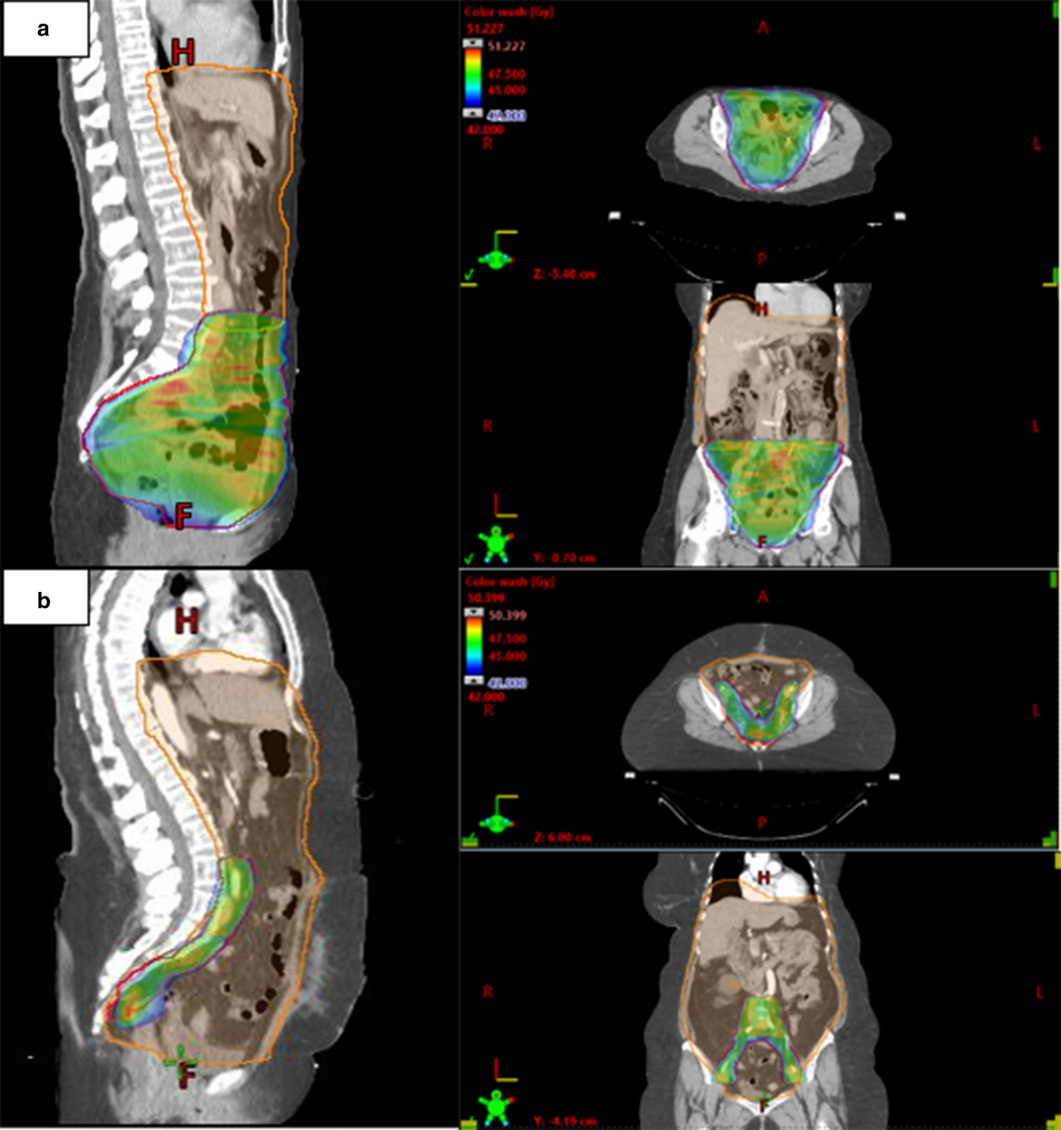
WART dose-volume distributions. Patients 1–4 (**a**) and Patient 5 (**b**). PTV_High (colored) confined to pelvis (**a**) and regional lymphatic compartment only (**b**). PTV_Low covers remainder of abdominal cavity

Patients were simulated and treated with an empty bladder. The patient was setup supine with arms-up using a combination Wing Board™ (Civco RT, Iowa) and pelvic BodyFix® cradle (Elekta AB, Sweden). A free-breathing planning 4-D CT was obtained without vascular contrast (except Patient 5) to capture full excursion of thoracic diaphragm and all intra-abdominal OAR motion. Patients were coached to replicate a smooth periodic respiratory motion. After CT acquisition, the entire parietal surface of the peritoneal cavity and including both thoracic and pelvic muscular diaphragms, was manually contoured as internal target volumes (ITV), expanded 5-10 mm, and subdivided into upper (or abdominal) low dose (PTV_Low) and lower (pelvic) high dose (PTV_High) planning target volumes. The level of the aortic bifurcation (Patients: 1–4) was chosen initially for this junction. Due to morbid obesity, Patient 5 had PTV_High confined to pelvic and distal para-aortic lymph nodes only (Fig. 1). Each PTV contained specific OAR structures which were separately contoured. The PTV_Low was expanded into the liver (10 mm) and kidney (5 mm) parenchyma as internal target margins to cover the peritoneal surface and account for any potential capsular infiltration. Validated normal tissue dose-volume tolerance parameters [32] were applied to the residual or “radiation-avoided” organ (i.e. kidney volume minus PTV_Low) and documented in Table 3. WART-specific PTV and OAR planning objectives were dosimetrically optimized within the treatment planning system (Eclipse™ v15.6, Varian Medical Systems, Palo Alto). Any dosimetric “hotspots” masked by the generated dose-volume-histogram (DVH) analysis were manually identified before treatment. Figure 1 represents a typical WART dose-wash (Patients: 1–4) and Patient 5.

**Table 3:** Summary of WART dose volume analysis

	Individual DVH parameters					Group DVH parameters				
	Patient 1	Patient 2	Patient 3	Patient 4	Patient 5	Min	Max	Median	Mean	**SD**
PTV_High										
D 95% (Gy)	43.40	44.30	43.60	42.49	43.62	42.49	44.30	43.60	43.48	0.65
D 98% (Gy)	42.54	43.50	42.90	41.38	42.63	41.38	43.50	42.63	42.59	0.77
D 2% (Gy)	49.71	48.40	48.10	49.47	48.53	48.10	49.71	48.53	48.84	0.71
D 1% (Gy)	49.99	48.60	48.30	49.77	48.77	48.30	49.99	48.77	49.09	0.75
D 50% (Gy)	47.08	46.80	46.70	46.59	46.33	46.33	47.08	46.70	46.70	0.28
Max	51.23	49.76	49.35	51.69	50.40	49.35	51.69	50.40	50.49	0.98
Min	36.26	38.61	23.49	34.71	35.88	23.49	38.61	35.88	33.79	5.93
Mean	46.79	46.52	46.36	46.28	46.17	46.17	46.79	46.36	46.43	0.24
Length: craniocaudal (cm)	18.60	20.40	18.40	11.40	16.80	11.40	20.40	18.40	17.12	3.44
PTV_Low										
D 95% (Gy)	21.74	20.60	17.70	22.94	20.95	17.70	22.94	20.95	20.79	1.95
D 98% (Gy)	21.07	19.40	17.30	22.02	19.43	17.30	22.02	19.43	19.84	1.81
D 2% (Gy)	47.34	46.90	33.70	39.86	40.90	33.70	47.34	40.90	41.74	5.63
D 1% (Gy)	48.34	47.50	38.50	46.27	42.61	38.50	48.34	46.27	44.64	4.07
D 50% (Gy)	25.50	26.10	25.80	26.38	26.43	25.50	26.43	26.10	26.04	0.39
Max	50.21	49.13	48.07	51.38	50.27	48.07	51.38	50.21	49.81	1.26
Min	15.50	13.45	16.25	14.85	11.18	11.18	16.25	14.85	14.24	2.00
Mean	26.47	26.55	25.15	26.91	26.76	25.15	26.91	26.55	26.37	0.70
Length: craniocaudal (cm)	30.00	22.80	20.00	32.80	42.00	20.00	42.00	30.00	29.52	8.70
Kidneys										
V 20 Gy (%)	52.27	50.50	44.60	26.44	4.98	4.98	52.27	44.60	35.76	20.02
Mean	19.87	18.53	17.40	17.52	14.40	14.40	19.87	17.52	17.54	2.02
D 2% (Gy)	25.05	26.53	26.40	24.56	20.96	20.96	26.53	25.05	24.70	2.26
D 1% (Gy)	25.30	26.79	26.67	25.17	21.46	21.46	26.79	25.30	25.08	2.16
Kidney L										
V 20 Gy (%)	50.26	51.90	44.10	31.41	6.50	6.50	51.90	44.10	36.83	18.77
Mean	19.70	18.69	17.32	17.22	14.70	14.70	19.70	17.32	17.53	1.88
D 2% (Gy)	26.07	26.72	26.34	24.80	21.22	21.22	26.72	26.07	25.03	2.25
D 1% (Gy)	25.16	26.99	26.52	25.24	21.64	21.64	26.99	25.24	25.11	2.10
Kidney R	54.49	49.00	45.10	36.55	3.13					
V 20 Gy (%)	19.99	18.37	17.50	18.94	14.16	3.13	54.49	45.10	37.65	20.38
Mean	25.01	26.26	26.50	25.80	20.43	14.16	19.99	18.37	17.79	2.22
D 2% (Gy)	25.30	26.48	27.69	26.22	21.04	20.43	26.50	25.80	24.80	2.51
D 1% (Gy)						21.04	27.69	26.22	25.35	2.55
Kidneys minus PTV										
V 20 Gy (%)	36.95	6.07	14.24	26.44	0.58	0.58	36.95	14.24	16.86	14.86
Mean	18.85	13.34	13.37	17.52	12.20	12.20	18.85	13.37	15.06	2.93
D 2% (Gy)	23.41	21.34	23.19	24.56	18.86	18.86	24.56	23.19	22.27	2.23
D 1% (Gy)	23.89	21.86	24.03	25.17	19.64	19.64	25.17	23.89	22.92	2.19
Bladder										
V 20 Gy (%)	100.00	100.00	100.00	100.00	99.00	99.00	100.00	100.00	99.80	0.45
V 40 Gy (%)	100.00	100.00	100.00	97.05	0.51	0.51	100.00	100.00	79.51	44.18
V 45 Gy (%)	98.24	99.00	99.70	92.83	0.00	0.00	99.70	98.24	77.95	43.66
Mean	47.43	47.32	46.73	47.00	27.18	27.18	47.43	47.00	43.13	8.92
D 2% (Gy)	49.22	48.79	47.76	49.58	35.99	35.99	49.58	48.79	46.27	5.79
D 1% (Gy)	49.36	48.94	47.87	49.79	38.30	38.30	49.79	48.94	46.85	4.83
Rectum										
V 20 Gy (%)	100.00	97.80	100.00	85.63	60.90	60.90	100.00	97.80	88.86	16.74
V 40 Gy (%)	95.49	79.90	98.30	70.18	17.84	17.84	98.30	79.90	72.34	32.57
V 45 Gy (%)	60.10	9.20	15.80	33.82	5.49	5.49	60.10	15.80	24.88	22.50
Mean	44.77	41.37	43.80	38.03	22.77	22.77	44.77	41.37	38.15	8.98
D 2% (Gy)	47.21	45.79	45.94	48.15	46.83	45.79	48.15	46.83	46.78	0.97
D 1% (Gy)	47.29	46.01	46.10	48.27	47.76	46.01	48.27	47.29	47.09	1.00
Liver										
V 20 Gy (%)	99.80	82.80	54.80	99.97	99.27	54.80	99.97	99.27	87.33	19.60
Mean	23.66	21.97	20.74	26.31	22.49	20.74	26.31	22.49	23.03	2.11
D 2% (Gy)	26.70	25.78	25.52	32.20	26.30	25.52	32.20	26.30	27.30	2.78
D 1% (Gy)	27.22	26.10	25.80	33.25	27.02	25.80	33.25	27.02	27.88	3.06
PTV_Total										
Length: craniocaudal (cm)	44.20	41.80	38.20	43.40	42.00	38.20	44.20	42.00	41.92	2.30
Isocentre Separation (cm)	15.00	10.00	14.00	20.00	15.00	10.00	20.00	15.00	14.80	3.56

PTV, planning target volume; D, dose; Gy, Gray; cm, centimetre; L, left; R, right; V, volume; Min, minimum; Max, maximum; SD, standard deviation

### Physics

Prior to treatment, all WART dosimetric plans were quality validated with point-dose small volume ion chamber measurements in a multi-modality tissue equivalent phantom device (CIRS Inc, Virginia). In WART body regions representative of PTV_High and PTV_Low and, also within the junctional overlap region between the respective isocentres, a homogeneity pass criteria of ± 3% was accepted. Delivered photon fluence (photons per measured surface area) was assessed with the collimator jaws of the linear accelerator (TrueBeam®, Varian Medical Systems, Palo Alto) restricted to the active area of the mega-voltage (MV) diode panel. Portal Dosimetry prediction with a gamma pass criteria of 3 mm/3% was deemed acceptable. Fluence was also validated pre-clinically with photon delivery to a dual orthogonal plane diode array (Delta4 phantom, Scanditronix, Sweden). Pre-implementation modeling of uncertainties of cranio-caudal isocentre positioning (i.e. superior or inferior drift) did not significantly perturb the dose distribution within the total peritoneal target areas (PTV_Low + PTV_High) as the WART plans were optimized with combined superior and inferior fields together (i.e. not a base plan then additional isocentre fields added ex post facto). The positional separation of the isocentres (10-20 cm) was initially chosen to maximize the overlap (and dosimetric homogeneity) between the 25 Gy and 45 Gy target regions whilst maintaining sufficient cranio-caudal range to cover the long combined PTV (up to 45 cm).

## Results

Median age of the study group was 58 years (range 47–68 years) and most (3/5) had documented severe pre-morbid endometriosis requiring at least one exploratory or emergency laparotomy over a period ranging 25 or 30 years before OCCC diagnosis. Overall 3 women had early stage OCCC: FIGO (2018) Stage I (IC1, IA, and IC3). The latter case (IC3) had positive peritoneal cytology as did 2 others with more advanced disease, 1 with implant metastases to the fallopian tube (IIA), and another with both extra-pelvic peritoneal dissemination and small volume para-aortic lymph node metastasis (IIIA2). Median [CA-125] at presentation was 56 kU/L (range 18.4–370 kU/L) which normalized (< 38 kU/L) before WART in all cases (Table 1).

Table 2 summarizes the IHC phenotypic signature of the study group. Patient 1 was the only subject with bilateral OCCC; histomorphological features were identical between both tumors and typical for OCCC, though the immunoprofile showed moderate aberrant WT-1 staining and only weak HNF1-β. This was the oldest case, and the only case with a large volume of tumor necrosis. Both the age of the paraffin blocks and the poor baseline tumour viability may have contributed to its immunoprofile. The latter may also be supported by slight asymmetry seen in the immunostaining, with moderate HNF1-β intensity seen primarily on the side with the smaller, better preserved, less necrotic tumor.

Median post-WART follow-up was 77 months (range 16–83 mo.). All patients remain clinically, biochemically, and metabolically (^18^FDG-PET) cancer-free to end August 2020.

### WART

Intensity modulated WART was completed as a consecutive 5-week out-patient program without interruption in all patients. A multi-arc dual isocentre VMAT technique was deployed consisting of 2 superior arcs which treated the abdomen (PTV_Low) and 2 inferior arcs treated the pelvis (PTV_High). Cranio-caudally the iso-centres were separated by 10–20 cm (mean 14.8 cm ± 3.56 (SD) cm). This separation enabled an extended (combined PTV) treatment length (PTV_Low + PTV_High) of between 38.20 to 43.20 cm (mean 41.92 cm ± 2.3 cm). Automatic couch movement accuracy (± 2 mm) limited measured dosimetric inhomogeneity to less than 5% within the PTV junctional zone.

**Fig. 2:**
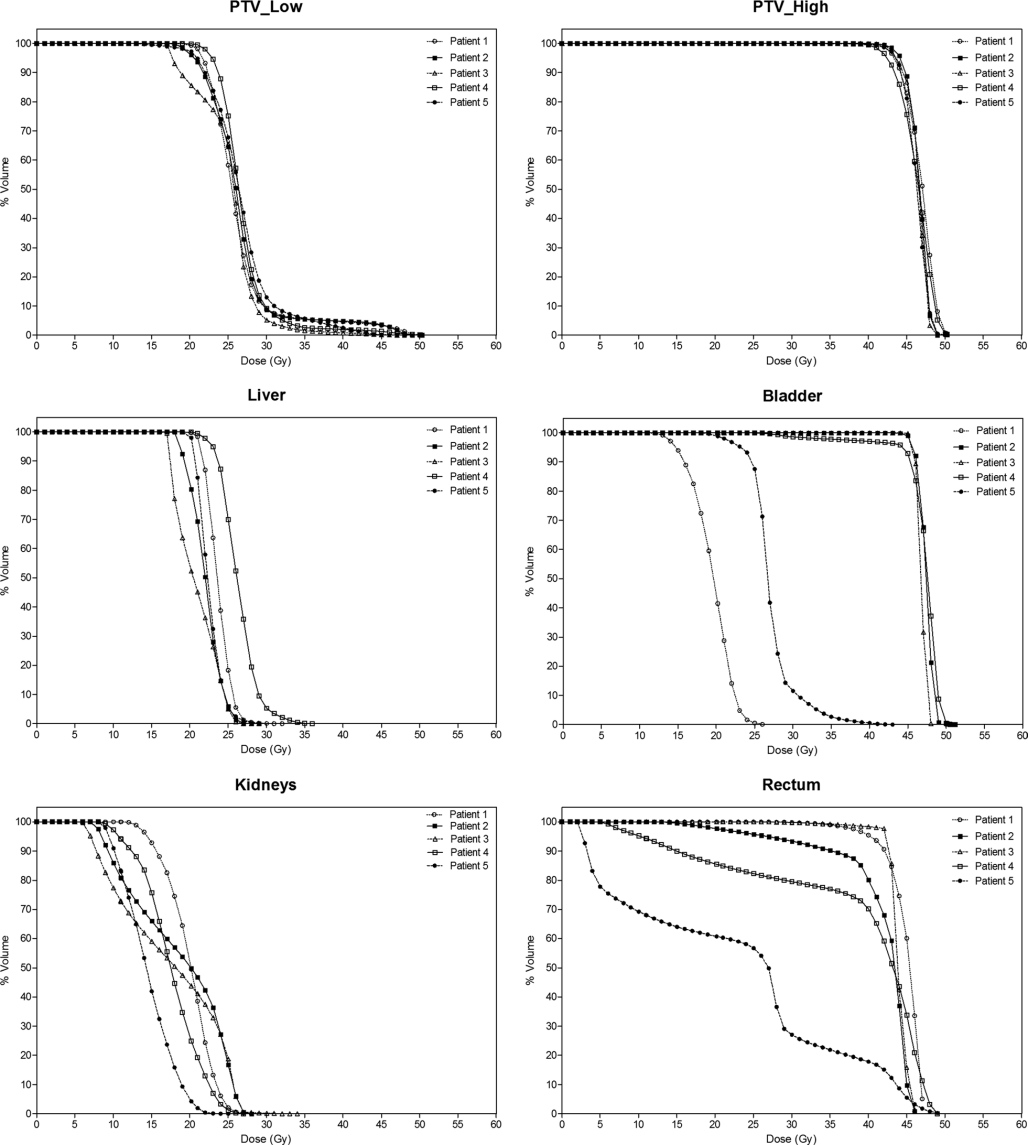
Individual and group DVH parameters

Evaluation parameters: A VMAT library of all 5 patients was created. Typical anatomical axial and coronal body dose wash and individual patient PTV dose-volume distributions and OAR exposures are depicted graphically in Fig. 2. Table 3 summarizes actual dose-volume-histogram (DVH) parameters for the PTVs and selected SOC-related OARs. Mean PTV_Low and PTV_High D95% coverage (dose to 95% of target volumes) were 20.9 Gy ± (SD) 2.0 Gy and 43.5 Gy (± 0.65 Gy), respectively. Mean liver and the combined kidney dose was 23 ± 19.6 Gy (SD) and 17.5 ± 2.0 Gy, respectively. Rectum (mean 38.2 ± 9 Gy) and bladder exposures (mean 43.1 ± 8.9 Gy) were dependent on inter-fractional (daily) filling with the respective organ volumes which received at least 45 Gy limited to 24.9% (± SD 22.5%) and 78% (± 43.7%). PTV_High in Patient 5 was personalized to include only the pelvic and para-aortic lymphatic compartments (Fig. 1).

Common acute toxicities included low grade persistent nausea, diarrhea and cysto-urethritis which required symptomatic management (all CTCAE < G3) for approximately 2–6 weeks post-WART.

**Fig. 3:**
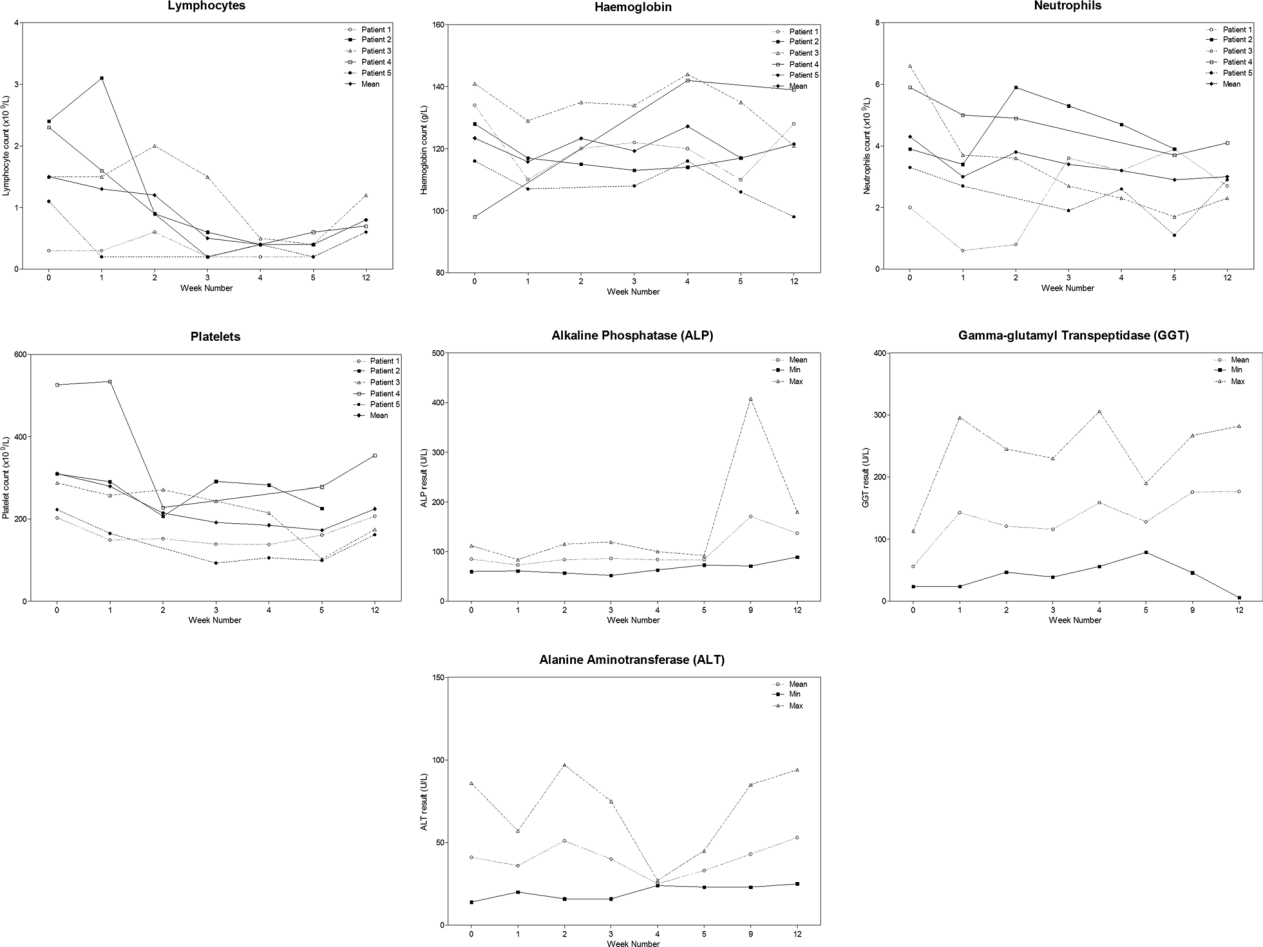
Hemologic and hepatic parameters

Figure 3 documents weekly fluctuations in mean hemoglobin concentration, total neutrophil and lymphocyte counts, and selected liver biochemistry during WART. There were no acute or post-week-12 perturbation in renal function (estimated GFR or serum creatinine; results not shown).

### Late toxicity

At week 12 post-WART, 3 CTCAE (v4.03) system organ classes (SOC) were re-assessed on blood parameters A low grade lymphopenia (median lymphocyte count 0.7; range 0.6–1.2 × 10^9^/L) persisted for about 6 months in 4 patients. Radiation-induced liver disease (RILD) produced minor asymptomatic elevations in the liver cholestasis enzymes (results not shown) more than 12 months post-WART. In 2 cases however, late musculo-skeletal SOC injury may have contributed to ALP elevation with low trauma skeletal insufficiency fractures of the irradiated lumbar spine (Patient 2) and sacro-iliac joint (Patient 4) as interval events detected on 12 month FDG-PET CT imaging. These were without clinical consequence and were associated with postmenopausal osteoporosis and responded to anti-resorptive therapy.

One patient (Patient 3) developed a severe (CTCAE G3) late radiation injury to the gastrointestinal tract SOC. Emergent entero-colitis was detected at 9 months post-WART and required elective major small bowel and colon resection. This woman had previously suffered significant symptomatic endometriosis for 25 years and had had a prior adnexectomy for an ovarian torsion in addition to multiple open laparotomies for bowel obstruction and adhesions over this period. At last follow-up 71 months post-WART the patient was feeding orally but had combined supplemental enteral feeding (percutaneous endoscopic gastrostomy: PEG tube) and a permanent intravenous catheter for parenteral nutritional support. She has regained her pre-treatment performance status, musculo-skeletal composition (on DEXA-scan), body weight (55 kg), and has returned-to-work.

## Discussion

Ovarian clear cell cancer (OCCC) was first recognized as a distinct sub-type of epithelial ovarian cancer by the World Health Organization in 1973 [33]. According to the dualistic model of epithelial ovarian carcinogenesis [7], OCCC is a Type I cancer which develops de novo from benign extra-ovarian Mullerian epithelial implants and is phylogenetically clustered with both endometrioid and seromucinous (mixed Mullerian) carcinomas as endometriosis-related cancers. Unlike the more prevalent Type II ovarian cancers (e.g., high grade serous), OCCC is considered genetically stable [7]. In this context, a genome-wide methylation and expression study in Japanese women has shown that OCCC maintains its malignant phenotype epigenetically through constitutive methylation, e.g. ER-α receptor and ARID1A (tumor suppressor gene) protein loss due to promoter hyper-methylation, and overexpression of the hepatocyte nuclear factor-1 (HNF-1) gene family due to promoter hypo-methylation (methylation loss) [34].

Within the major HNF-1 transcription network, HNF-1ß protein expression is almost ubiquitous in OCCC and uniquely characterizes its classical phenotype [31, 35]. Putatively, HNF-1ß drives chemotherapy drug resistance through numerous down-stream mechanisms including increased drug efflux, enhanced nucleotide excision repair (NER), increased drug detoxification, and cell cycle control (maintenance of TP53-wild type) [2, 4–6, 16, 36]. Although significant subsets (20–40%) of OCCC also manifest defective homologous recombination repair (HRR), micro-satellite instability, and harbor potentially targetable deleterious activating mutations of regulatory pathways (e.g. phosphatidylinositol 3-kinase [PI3K/AKT/mTOR], receptor tyrosine kinase [RTK/RAS], etc.), no reliable clinical treatment strategies have thus far emerged [11, 36, 37]. In a novel Phase II Gynecologic Oncology Group study, for example, advanced stage OCCC patients had no significant increase in progression-free survival when the standard doublet of carboplatin and paclitaxel was combined with the mammalian target of rapamycin (mTOR) inhibitor, temsirolimus [35]. Further, the predicted survival advantage of a non-cross resistant dose dense platinum/irinotecan combination was not seen in a recent randomized Japanese Phase III study [38]. Multiple pleiotropic OCCC resistance pathways have collectively limited many of the current and potentially translatable systemic therapeutic paradigms.

We have demonstrated for the first time the clinical utility of adjuvant intensity-modulated whole abdominal radiation therapy (WART) in predominantly early stage optimally de-bulked OCCC with harmonious histo-morphic and phenotypic signatures which over-expressed HNF-1ß. At a median follow-up of 77 months, all treated women in our small cohort remain clinico-radiologically, and biochemically (CA-125) cancer-free with good self-reported quality of life.

Late gastro-intestinal toxicity remains the dominant serious emergent hazard of WART [3, 19, 21, 24]. In this series, elective bowel resection and long-term supplemental nutritional support was required in one patient with advanced stage OCCC and multiple prior endometriosis-related laparotomies. Re-defining the high dose volume (PTV_High) posteriorly to the retro-peritoneal pelvic and aortic lymph node spaces only (as in Patient 5; Fig. 1) would reduce the uncertainty within the high-dose PTV junctional zone which invariably contains the at-risk bowel. Conceptually, a homogeneous total WART dose of 22–25 Gy delivered over 4 weeks to the entire parietal peritoneum would probably suffice (with PTV_High confined to regional lymph nodes) in early stage patients with positive peritoneal cytology and no residual pelvic disease burden. Data from the intra-peritoneal radio-phosphorous (^32^P) era are provocative in this regard [3]. Unlike radio-phosphorous however, intensity modulated WART can also target the extra-peritoneal lymphatic compartment within a personalized PTV_High in women predicted to have at-risk bowel (e.g. multiple prior laparotomies, or extensive adhesions noted at initial de-bulking surgery).

Two patients were detected with asymptomatic (CTCAE G2) fractures of the sacrum and lumbar vertebra on planned FDG-PET surveillance at 1-year. Osteoporotic fragility fractures are probably under-reported in post-menopausal women following pelvic and extended pelvic irradiation for gynecologic cancer [22, 40].

Adjuvant WART for epithelial ovarian cancer is not new. Developed in North America and Canada in the pre-platinum era (1960–1985), WART was once a transformative treatment with real strategic intent for the management of ovarian cancer [3, 24]. Prospective observational and randomized studies at The Princess Margaret Hospital (PMH) [19, 21, 24] developed the classic “Boolean” decision algorithm of: FIGO stage, residual disease burden (none versus < 2 cm), histologic grade (1–3), and epithelial ovarian subtype, to define an intermediate-risk group of FIGO Stage I-III patients with an impressive 70% 5-year relapse-free survival after WART. The PMH technique was shown to generalizable to pure OCCC cases in 2007 [20]. This Japanese matched-pair analysis included 28 advanced stage patients (up to 20 mm pelvic residuum) and intentionally excluded women with favorable (Stage IA/B) disease. Actual 5-year overall and progression-free survival compared to women given platinum-based chemotherapy significantly favored WART: 81.8% and 81.2% versus 33% and 25%, respectively. Other groups [22, 23, 25] have externally validated the clinical utility of adjuvant first-line, and consolidation (post-chemotherapy) WART in all epithelial ovarian subtypes, including OCCC. Disappointingly, the most recent PMH publication [41] failed to confirm the efficacy of WART over chemotherapy, or no further therapy, in a large retrospective review of 163 OCCC cases treated over a 20-year period (1995–2014). However, analysis of this report revealed that only 27% (44/163 women) had any form of radiation therapy and almost half (20 women) were prescribed pelvic radiation either alone or as post-chemotherapy consolidation. WART as a definitive mono-therapy was described in only 7 patients overall (4%), rendering any efficacy conclusions unsafe.

Historical WART techniques consisted of large anterior and posterior radiation portals as either “opposed open-field” (or classic PMH technique [19]), or manual “moving strip” (MD Anderson Cancer Centre, Houston [3]). These early techniques lacked the sophistication of VMAT particularly 4-D real-time adaptive respiratory motion management, modulation of radiation intensity during delivery, and on-line CT image-guidance to verify dose-volume coverage and OAR avoidance.

Despite the short-comings, WART has improved long-term progression-free and overall survival in optimally de-bulked Stage I-III ovarian cancer patients [19]. Safety signals associated with acute GI toxicity and emergent late radiation bowel and skeletal injury need consideration however. Concomitant or sequential use of chemotherapy with WART, and combined intra-peritoneal colloidal ^32^P and whole pelvis irradiation in historical series [3, 21–23, 25], were known to amplify the risk for serious late toxicity. Post-WART bowel injury was strongly correlated with adhesions induced from extended lymph node dissection, previous surgeries for complicated endometriosis (as in our Patient 3), and the routine second-look laparotomy (SLL) which framed earlier ovarian cancer care-standards.

The strengths and limitations of pragmatic observational studies to inform clinical decision making have been recently outlined in a research statement by the American Society of Clinical Oncology [43]. As demonstrated by our small study, valid empirical evidence can be generated from such analyses with potential for real-world applicability and low cost. An inherent strength of observational research is hypothesis generating. Our novel hypothesis that HNF-1ß expression may portend drug resistance needs confirmation and may influence future care standards.

The internal validity of our study was further strengthened by the treatment of a histologically harmonious cohort of OCCC patients with full adherence to a conceptually uniform WART protocol, and standardized follow-up assessments. Our WART database was enhanced by curation of radiation target volumes and critical avoidance organs to the AAPM Task Group 263 nomenclature [30]; this was done to promote future transparency for “Big Data” analysis as definitive evidence of WART utility in a rare cancer such as OCCC, will probably be only be derived from multi-institutional observational research [44]. Weaknesses of our study include small sample size, lack of a comparison group, and selection bias favoring early stage patients with potential over-estimation of WART treatment effect. Additionally, WART was personalized to body habitus and predicted toxicity risk in one case (Patient 5), which may have weakened overall generalizability.

## Conclusions

In conclusion, we believe intensity modulated WART has excellent clinical utility in optimally de-bulked women with histologically confirmed pure OCCC whose phenotypic signature includes HNF-1β over-expression. Rigorous future observational research malleable to “Big Data” collaboration is needed to enhance the evidence-base supporting this imminently actionable radiation technique.

## Data Availability

The datasets used and/or analysed during the current study are available from the corresponding author on reasonable request.

## Abbreviations

AAPM: American Association of Physics in Medicine
AMACR: Alpha methyl Acyl-CoA racemase
ARID1A: AT-rich interaction domain 1A
CT: Computed tomography
CTCAE: Common terminology criteria for adverse events
ER-α: Estrogen receptor-alpha
FBC: Full blood count
^18^FDG-PET: ^18^Fluoro-deoxyglucose positron emission tomography
FIGO: Federation of gynecology and obstetrics
HNF: Hepatocyte nuclear factor
HRR: Homologous recombination repair
IARC: International Agency for Research on Cancer
IHC: Immuno-histochemical
ITV: Internal target volumes
NER: Nucleotide excision repair
NSLHD: Northern Sydney Local Health District
OAR: Organ at risk
OCCC: Ovarian clear cell cancer
PTV: Planning target volume
PTV_High: Planning target volume high dose
PTV_Low: Planning target volume low dose
SOC: System organ classes
VMAT: Volumetric modulated arc therapy
WART: Whole abdominal radiation therapy
WT-1: Wilm’s tumor suppressor
